# Self-Healing Properties of Water Tree Damage in Multilayered Shell–Core-Structured Microcapsules/Cross-Linked Polyethylene Composites

**DOI:** 10.3390/polym16010155

**Published:** 2024-01-04

**Authors:** Bo Zhu, Hao Sun, Yaqi Zhu, Shengkun He, Ximu Han

**Affiliations:** MOE Key Laboratory of Engineering Dielectrics and Its Application, Harbin University of Science and Technology, Harbin 150080, China

**Keywords:** multifunctional composites, nanoparticles, electrical properties, microstructures

## Abstract

To investigate the effect of the structure of microcapsules on the properties of cross-linked polyethylene (XLPE) composites, three XLPE specimens filled with multilayered shell–core-structured microcapsules are designed. In this paper, the microcapsules are first analyzed morphologically and chemically. In addition, the effect of the microcapsule structure on the typical electrical properties of the composites is explored. Finally, the self-healing ability of XLPE specimens filled with microcapsules is verified. The results show that the SiO_2_ on the surface of the trilayer shell–core microcapsules can make the microcapsules and the XLPE matrix have a better mechanical interlocking ability, which makes the typical properties of the trilayer shell–core microcapsules slightly better than those of the bilayer shell–core microcapsules. Moreover, when the bilayer shell–core or trilayer shell–core microcapsules rupture under the action of an electric field, the repair material reacts with the water tree under capillary action to consume the residual water while generating organic matter to fill in the cavity, thus repairing the damaged area of the water tree and ultimately achieving the self-healing of the composite water tree.

## 1. Introduction

In recent years, with the development of society and China’s national economic level, the demand for electricity in people’s livelihoods and all walks of life continues to rise. The cable plays a transmission role, an essential part of the power system [1,2]. XLPE cables are widely used in urban power grids because of their reliable electrical and mechanical properties. XLPE power cables have the advantages of high breakdown field strength, high allowable working temperature, high current-carrying capacity, ease of laying and installation, and low transmission loss. Low-density polyethylene is cross-linked and modified by the cross-linking agent. The peroxide cross-linking agent decomposes under the action of heat to generate free radicals, and these free radicals seize the hydrogen atoms on the polyethylene chain to turn them into active polyethylene macromolecule free radicals, which generates carbon–carbon cross-linking and forms a planar mesh structure. The specific cross-linking reaction process is shown in Equations (1)–(3) below.

(1) $$\mathrm{ROOR}\to 2R\dot{O}$$

(2) $$∼{\mathrm{CH}}_{2}—{\mathrm{CH}}_{2}—{\mathrm{CH}}_{2}—{\mathrm{CH}}_{2}∼+R\dot{O}\to ∼{\mathrm{CH}}_{2}—{\mathrm{CH}}_{2}—\dot{C}H—{\mathrm{CH}}_{2}∼+\mathrm{ROH}$$

(3) $$\begin{array}{ccc} {2\sim \mathrm{CH}}_{2}—{\mathrm{CH}}_{2}—\dot{C}H—{\mathrm{CH}}_{2}\sim & \to & {\sim \mathrm{CH}}_{2}—{\mathrm{CH}}_{2}—\mathrm{CH}—{\mathrm{CH}}_{2}\sim \\ & & |\\ & & {\sim \mathrm{CH}}_{2}—{\mathrm{CH}}_{2}—\mathrm{CH}—{\mathrm{CH}}_{2}\sim \end{array}$$

However, the production and operation of the line are affected by factors such as electricity, heat, and mechanical stress, which will inevitably produce microscopic defects. These tiny defects are gradually induced by water erosion, which reduces the insulation performance of the cable [3,4,5]. Water tree defects in solid insulation are a permanent material degradation phenomenon that occurs inside the insulation. A water tree is a dendritic dispersion of insulation defects formed by micron-sized holes and nanoscale channels. Under high electric fields, moisture gradually accumulates in the XLPE material, forming water tree channels. Water tree channels lead to a decrease in the dielectric strength of the material and an increase in the electrical resistance, which in turn causes partial discharges and material damage. Although there have been techniques to repair water dendrite defects in cables by injecting a repair agent that reacts with water, the method requires the line to be de-energized, pressurized, among others, which severely limits the scope of its use in a practical sense [6]. At the same time, due to the difference between the molecular diffusion rate of the restoration solution and the catalyst, resulting in the initial position being prone to the accumulation of the restoration solution and becoming clogged, the effect of the water tree inside the cable restoration is not as expected [7,8]. Therefore, XLPE materials need to have a self-healing function that is capable of autonomously repairing areas of water tree damage to extend the service life of the cable.

Microencapsulation is a technology that separates the contents of a capsule from its surroundings and achieves compatibility with specific materials by forming a thin protective layer around solid particles, droplets, or bubbles [9,10]. When the repaired material is damaged, the microcapsule ruptures under certain conditions, and the internal repair material is released to fix the defective structure, thus restoring the material properties to a certain extent [11]. Therefore, the viscosity of the repair solution must be low enough to allow an easy flow out of the ruptured capsule within a reasonable time, followed by the repair of the damaged area and the filling of the cavity [12]. The shell wall of the microcapsules should be constructed well and strong enough to withstand the impact of the core material [13]. But, at the same time, it should be sufficiently fragile to break under specific conditions to satisfy the triggering requirement, thereby releasing the core material when a defect occurs [14]. When the XLPE material undergoes water tree aging, the microcapsules rupture under the action of the electric field, and the repair material reacts with the water in the water tree branches under the action of the catalyst to produce organic matter. Therefore, to ensure the structural stability of the XLPE filled with microcapsules, the shell wall of the microcapsule should have a good compatibility and adhesion with the surrounding polymer matrix. The restorative material must be mixed quickly and have low shrinkage during polymerization [15]. The repair solution should be cured slowly. There should be enough time to fill the cracks, and the fixed healing agent should have good adhesive properties (to rebind the cracked surface) and mechanical properties [16,17]. At present, most domestic and international analyses of microcapsule self-repairing technology have focused on the effect of single-structure microcapsules on the performance of matrix materials [18,19,20]. However, there are few reports on the effects of multilayered shell–core structures on the performance of the matrix. Therefore, in this paper, we fabricate three microcapsules with multilayered shell–core structures to investigate the impact of the various structural properties of the microcapsules on the performance of the XLPE composites in terms of electrical properties and self-repairing effects.

This work focuses on the study of the effect of the structure of the microcapsules on the properties of the XLPE composites. Thus, the research uses interfacial polymerization to prepare three microcapsules with different shell and core structures by selecting DTMS and LABSA as the core material and nano-SiO_2_ surface-modified UF as the wall material. Furthermore, the preparation of XLPE specimens filled with microcapsules is investigated, in which the content of the microcapsules with three different structures was 1.0 wt%. Moreover, the typical characteristics of XLPE specimens filled with microcapsules are investigated according to parameters such as the crystallinity, break-down field strength, dielectric properties, and space charge of the composites. The change mechanism of each property is analyzed to obtain the optimal microcapsule doping structure of the XLPE composites. Meanwhile, the self-healing ability of the microcapsules for water tree aging is demonstrated using water tree aging experiments on XLPE specimens filled with multilayered shell–core-structured microcapsules.

## 2. Experimental Procedure

### 2.1. Selection of Materials and Experimental Equipment

Urea and ammonium chloride were obtained from Sinopharm Chemical Reagent Co. (Shanghai, China) Formaldehyde solution, triethanolamine (TEA), resorcinol, and octanol were obtained from Tianjin Damao Chemical Reagent Factory (Tianjin, China). Dodecyltrimethoxysilane (DTMS) was obtained from Shanghai Eon Chemical Technology Co. (Shanghai, China), and the chemical formula is C_15_H_34_O_3_Si, and was the repair material used for water tree restoration. Linear alkyl benzene sulphonate (LABSA) was obtained from Shandong Yusuo Chemical Technology Co. (Heze, China), and the chemical formula is C_18_H_30_O_3_S, and acted as a catalyst in the reaction between the restorative solution and water. Sodium dodecyl benzene sulfonate (SDBS) was obtained from Tianjin Beichen Fangzheng Reagent Factory (Tianjin, China), and the chemical formula is C_18_H_29_NaO_3_S, and was used as an emulsifier in the process of microcapsule encapsulation and moulding. The above reagents were all of AR (analytically pure) grade. Low-density polyethylene (LDPE) was obtained from Sinopec Beijing Yanshan Branch (Beijing, China), and the chemical formula is (C_2_H_4_)_n_, which is a kind of insulating material. Dicumyl peroxide (DCP) was obtained from Shanghai Gaoqiao Chemical Co. (Shanghai, China), and the chemical formula is C_18_H_22_O_2_, and acted as a cross-linking agent in the preparation of XLPE. The antioxidant 1010 was obtained from Hebei Baiyilian Chemical Co. (Shijiazhuang, China).

### 2.2. Preparation of the Microcapsules with Multilayered Shell–Core Structures

The fabrication of the microcapsules with multilayered shell–core structures was divided into the following four steps:

Step 1. Preparation of a UF prepolymer coated with nano-SiO_2_: After mixing and stirring the nano-SiO_2_ and water containing 37% of formaldehyde, urea dissolved in deionized water was added, and the pH of the system was adjusted to 8~9 with TEA. The reaction was held at 70 °C for 60 min and cooled to room temperature. The resulting UF prepolymer coated with nano-SiO_2_ was a white and somewhat viscous solution [21].

Step 2. Production of the monolayer shell–core microcapsules: Under the condition of a 40 °C water bath, the emulsifiers SDBS, LABSA, and deionized water were mixed and stirred for 30 min. When the solution appeared milky white, we raised the pH of the solution to 9~10 slowly using a sodium hydroxide solution, and ammonium chloride, resorcinol, and UF prepolymers were added sequentially and dropwise. After that, the pH of the solution was slowly lowered to about 3.0 using citric acid, and after the reaction was completed by holding and stirring at 53 °C for 3 h, it was filtered, washed, and dried to obtain a sample of microcapsules with a monolayer shell–core structure in powder form. The wall material of the monolayer shell–core microcapsules was urea–formaldehyde resin, and the core material was the LABSA catalyst.

Step 3. Fabrication of the bilayer shell–core microcapsules: After mixing and stirring the emulsifiers SDBS, DTMS, monolayer shell–core microcapsules containing catalysts, and deionized water for 30 min at 40 °C in a water bath, the unsurface-modified UF prepolymers, ammonium chloride, and resorcinol were added dropwise in sequence. The pH of the solution was slowly adjusted to about 3.0 with diluted hydrochloric acid and then warmed up to 53 °C, and the reaction was held and stirred for 3 h. After filtration, washing, and drying, a UF-DTMS/LABSA microcapsule sample with a bilayer shell–core structure was obtained. The wall material of the bilayer shell–core microcapsules was urea–formaldehyde resin, and the core material consisted of the monolayer shell–core microcapsules and the DTMS repair material.

Step 4. Production of the trilayer shell–core microcapsules: Under a 40 °C water bath, the SDBS emulsifier, DTMS, the monolayer shell–core microcapsules containing catalysts, and deionized water were mixed and stirred for 30 min. Ammonium chloride, resorcinol, and UF prepolymer modified with a nano-SiO_2_ surface were added dropwise. The pH of the solution was slowly reduced to about 3.0 with diluted hydrochloric acid and then heated to 53 °C. After holding and stirring for 3 h, the UF@SiO_2_-DTMS/LABSA microcapsule sample with a trilayer shell–core structure was obtained after filtration, washing, and drying.

**Figure 1:**
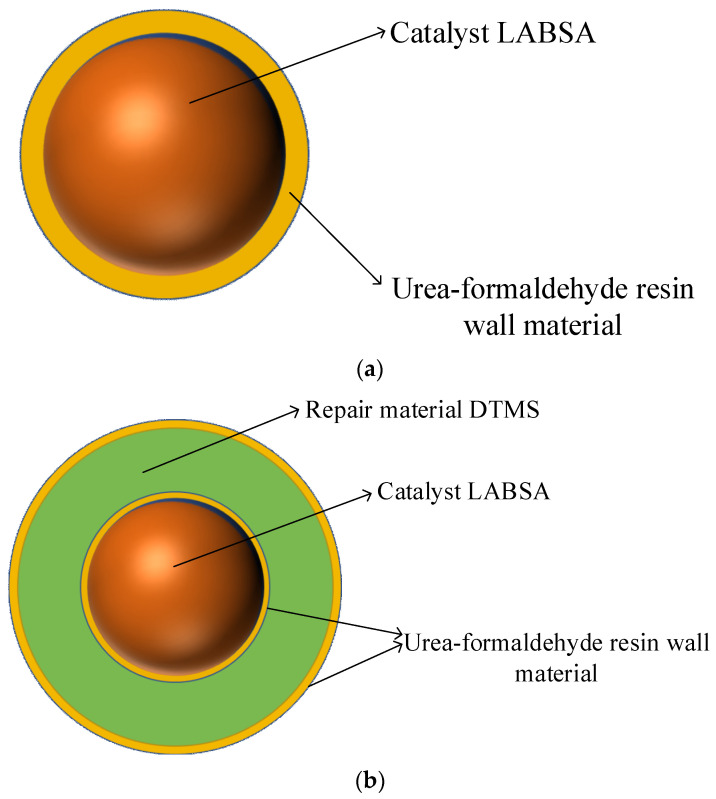

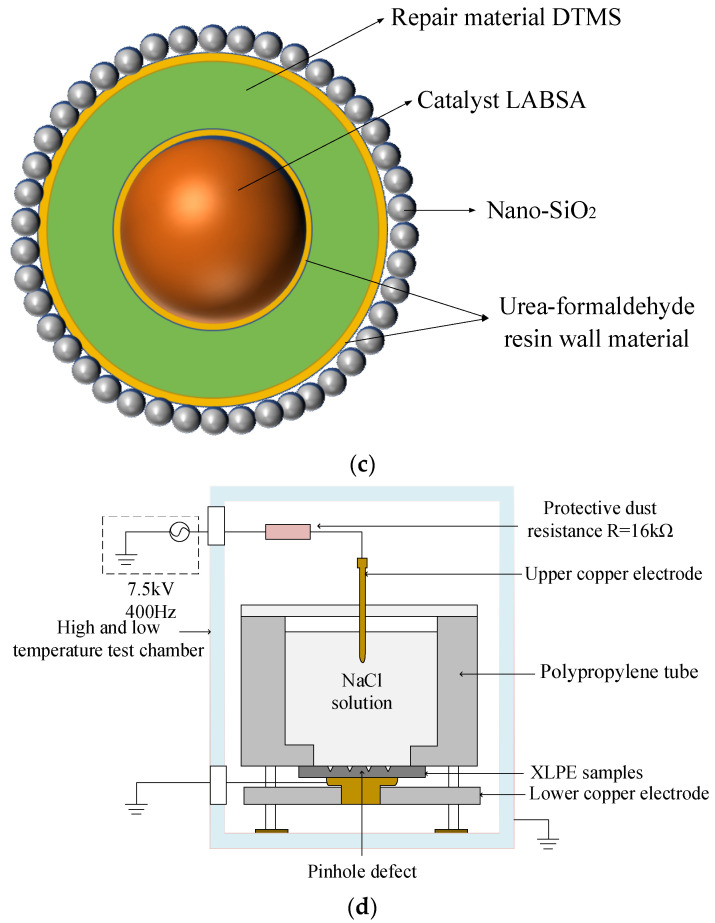
(**a**) Internal structure of the monolayer shell–core microcapsules. (**b**) Internal structure of the bilayer shell–core microcapsules. (**c**) Internal structure of the trilayer shell–core microcapsules. (**d**) Schematic diagram of the water tree aging experimental device.

Figure 1c illustrates the internal structure of the trilayer shell–core microcapsules. It can be seen from Figure 1c that nano-SiO_2_ exists on the surface of the urea–formaldehyde resin with trilayer shell–core microcapsules. To ensure that the siloxane restoration solution can be fully hydrolyzed and condensed with the water in the water tree, the mass ratio of siloxane to catalyst in the microcapsule was about 4:1. At the same time, the dosage of ammonium chloride and resorcinol accounted for 1% and 2% of the mass of the UF prepolymer, respectively, to ensure the structural stability of the microcapsule.

### 2.3. Preparation of XLPE Specimens Filled with Multilayered Shell–Core-Structured Microcapsules

The LDPE was placed in the torque rheometer (Harbin, China); when the LDPE was in the molten state, and the torque was smooth, the antioxidant, microcapsules, and DCP were placed in order, and the mixing time was 20 min in total. After completion, the homogeneous mixture of LDPE, antioxidants, microcapsules, and DCP was taken out and cut into small pellets for subsequent use. The concentrations of antioxidants, microcapsules, and DCP were 0.3 wt%, 1.0 wt%, and 1.8 wt%, respectively. The role of DCP is to improve the strength and elasticity of the material by the cross-linking reaction to entangle the LDPE molecular chains with each other, resulting in C-C chemical bonding and forming a mesh structure. In addition, adding antioxidants to the insulating materials can reduce the damage in the process of the thermal oxygen aging of materials, and it also has a certain inhibitory effect on the water tree aging of cables.

The blended composites were sequentially melt-molded in a plate vulcanizing machine (Huzhou, China) at 110 °C and cross-linked in a plate vulcanizing machine at 175 °C and 15 MPa for 35 min, after which the obtained XLPE composites were sufficiently cooled and vacuum-dried to obtain XLPE specimens filled with multilayered shell–core-structured microcapsules.

### 2.4. Characterization

In this paper, the effects of microcapsules with multilayered shell–core structures on the insulation strength of the XLPE materials were characterized using DSC (differential scanning calorimetry), SEM (scanning electron microscope), OM (optical microscope), AC breakdown field strength, space charge properties, and dielectric properties. Moreover, the mechanism of the microcapsule system on the electrical properties of the XLPE materials was explored.

XLPE is an insulating material with an internal crystalline structure and amorphous regions. The current electro-mechanical damage theory of water tree growth suggests that the water tree growth is mainly concentrated in the amorphous region of the material. Therefore, the degree of crystallinity can directly affect the material’s various properties and the water tree’s development trend. In this study, the DSC (differential scanning calorimetry) method was used to test the absorptive and exothermic temperature spectra and calculate the crystallinity of the XLPE composites with multilayered shell–core-structured microcapsules. The temperature range was 25 °C~150 °C, the heating/cooling rate was 10 °C/min, and the nitrogen flow rate was 150 mL/min. The mass of the specimens used was 5~10 mg.

The breakdown field strength is one of the key indicators to characterize the macroscopic properties of materials, which is the critical ability of materials to maintain the insulating state under an applied electric field. The insulation strength of the composite material was measured using an AC experimental platform (Harbin, China). The diameter of the high-voltage electrode used was 25 mm, and the thickness of the specimens was about 200 μm. The whole container was filled with dimethyl silicone oil, which was required to submerge the high-voltage electrode and the entire sample. During the experiment, the voltage was raised slowly at the same speed; when the breakdown occurred, the voltage was quickly stopped, and the over-current reset was carried out. The corresponding voltage indication (breakdown voltage of the corresponding thickness) was recorded, the specimens were replaced, and the above operation was repeated until 10 data were obtained for each group of samples.

The relative dielectric constant is a macroscopic constant that characterizes the polarization strength of the material under an alternating electric field. In contrast, the dielectric loss factor accounts for the loss of the material during polarization. In this paper, dielectric measurements (Harbin, China) were conducted to analyze the dielectric properties of the materials, and the results obtained can be divided into relative permittivity (real part) and dielectric loss factor (imaginary part). The temperature of the experiment was room temperature, and the frequency range of the measurement was from 10^−1^ to 10^6^ Hz.

The space charge characteristics of XLPE specimens filled with microcapsules were measured using a space charge acquisition system (Shanghai, China), which was designed based on the principle of pulse electroacoustic method (PEA) testing. The temperature of the experiment was room temperature, the test field strength was 48 kV/mm, and the pressurization time was 3600 s. Moreover, dimethyl silicone oil was applied between the electrode and the specimen to eliminate the effect of the air gap on the experimental results and to ensure the accuracy of the experiment.

### 2.5. Accelerated Water Tree Aging Experiment

In order to accelerate the water tree growth rate of the XLPE composites, this experiment used the water needle electrode method to conduct an accelerated water tree aging experiment using XLPE materials. A square XLPE specimen with a thickness of 4 mm and a side length of 6 cm was fabricated using the method described in Section 2.3. Three rows of pinhole defects were made with a steel needle in the area directly in the center of the specimen, with about 6 to 8 pinholes spaced at a distance of 3mm in each row. A 20% sodium chloride solution was injected into the polypropylene tube and inserted into the copper electrode, after which an accelerated water tree aging test was conducted for 30 days. The virtual value of the power supply used in the experiment was 7.5 kV, the frequency was 400 Hz, and the waveform was sinusoidal. The protection resistor resistance value was 16 kΩ. Figure 1d shows the schematic diagram of the XLPE cable water tree aging experiment.

### 2.6. Measurement of the Depolarizing Current using the PDC Method

In this paper, the PDC (polarizing–depolarizing current) method was used to measure the polarization/depolarization currents of the aging specimen. Since the measured currents were at the pA level, which was a weak current, the PDC circuit was based on a three-electrode system (Harbin, China) to eliminate the effect of the leakage currents. At the same time, in order to shield the external electromagnetic interference, it was necessary to place the main control part of the test circuit and the tested cable in the closed distribution box respectively, and the distribution box shell was grounded. The experimental circuits were all grounded at a single point to prevent the weak current backflow in the ground from affecting test accuracy. In addition, the current data collected in the first 5 s were removed when performing the PDC analysis so as to reduce the errors generated by the experiment.

## 3. Results and Discussion

### 3.1. Characterization of the Microcapsules with Multilayered Shell–Core Structures

#### 3.1.1. Microscopic Morphology of the Microcapsules with Multilayered Shell–Core Structures

**Figure 2:**
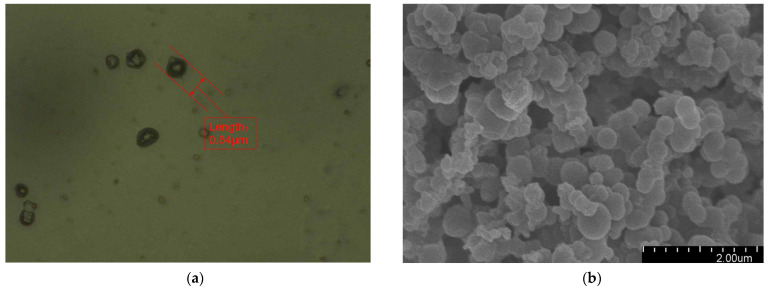

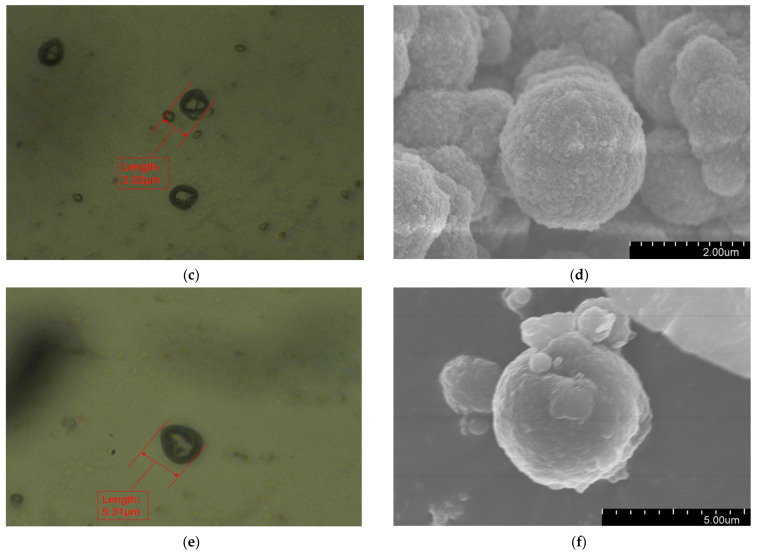
Microscopic morphologies of the microcapsules with multilayered shell–core structures: (**a**) OM photograph of the monolayer shell–core microcapsule; (**b**) SEM photograph of the monolayer shell–core microcapsule; (**c**) OM photograph of the bilayer shell–core microcapsule; (**d**) SEM photograph of the bilayer shell–core microcapsule; (**e**) OM photograph of the trilayer shell–core microcapsule; (**f**) SEM photograph of the trilayer shell–core microcapsule.

**Figure 3:**
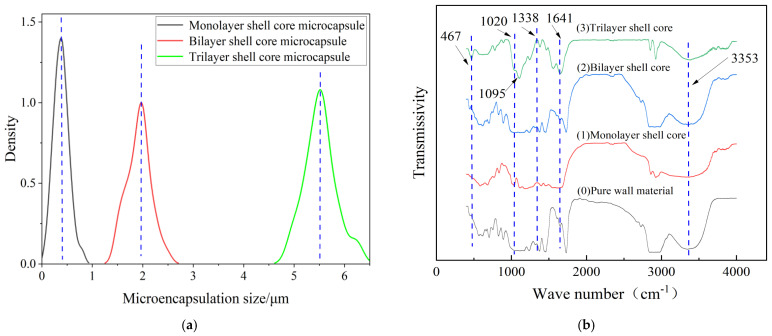
(**a**) Particle size distribution of microcapsules; (**b**) infrared spectra of microcapsules with multilayered shell–core structure.

Figure 2 presents the morphology of the fabricated microcapsules with multilayered shell–core structures observed using OM (Harbin, China) and SEM (Harbin, China). The morphology of the prepared microcapsules was spherical particles with a rough outer surface and no obvious breakage. This morphology resulted in a better mechanical interlocking ability between the microcapsules and the XLPE matrix. By statistically organizing the microscopic morphology of the microcapsules, 50 microcapsule sizes were selected to obtain the particle size distribution of the microcapsules, and the results are shown in Figure 3a. The average diameter of the monolayer shell–core microcapsules was stabilized at about 0.4 μm, the average diameter of the bilayer shell–core microcapsules was stabilized at about 2 μm, and the average diameter of the trilayer shell–core microcapsules was stabilized at about 5.5 μm, which proved that the prepared microcapsules had a multilayered shell–core structure.

#### 3.1.2. Chemical Characterization of Microcapsules with Multilayered Shell–Core Structures

This paper used the FT/IR−6100 optical path Fourier spectrometer (Shanghai, China) to test the chemical structures of the microcapsules to verify the validity of the microcapsule preparation process. The tested wave number range was 400−4000 cm^−1^, and the results of the IR spectral analysis of microcapsules with different shell–core structures are shown in Figure 3b.

Among them, 467 cm^−1^ was the vibrational absorption peak of Si−O, 1020 cm^−1^ was the C−O vibrational absorption peak due to the primary alcohols produced during the hydroxy-methylation of UF, and 1641 cm^−1^ was the C=O vibrational absorption peak. In addition, the broad and strong vibration absorption peak at 3353 cm^−1^ was the result of the overlapping of N−H and O−H, 1095 cm^−1^ was the vibrational absorption peak of Si−O−Si, and 1338 cm^−1^ was the vibrational absorption peak of S=O. It can be seen that there is a prominent infrared characteristic absorption peak at 467 cm^−1^ for the bilayer shell–core microcapsule and trilayer shell–core microcapsule specimens, which indicates the presence of the siloxane restorative fluid DTMS within the microcapsules. Furthermore, all three microcapsules show a characteristic absorption peak at 1338 cm^−1^, indicating the presence of the catalyst LABSA inside the microcapsules of all three structures. The trilayer shell–core microcapsule specimens showed a characteristic absorption peak at 1095 cm^−1^, indicating the presence of nano-SiO_2_ on the surface of the microcapsules. Therefore, the infrared spectra of the multilayered shell–core-structured microcapsules proved the effectiveness of the abovementioned microcapsule preparation process.

### 3.2. Characterization of Self-Healing Composites

#### 3.2.1. Crystal Melting Characteristics

**Figure 4:**
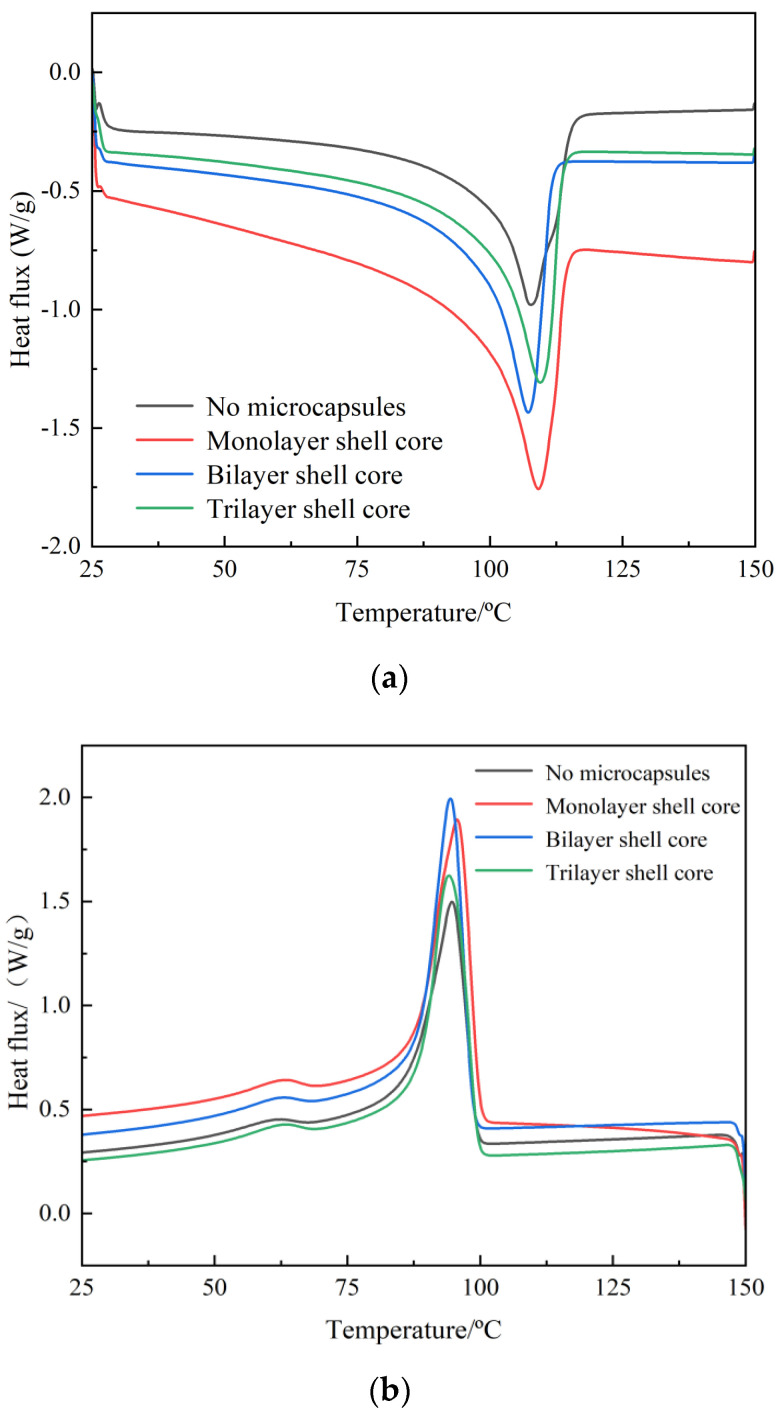
DSC temperature spectra of XLPE specimens filled with multilayered shell–core–structured microcapsules: (**a**) the melting process; (**b**) the crystallization process.

In this paper, DSC test of composite materials was carried out by differential scanning calorimeter (Chengdu, China). The DSC test results of XLPE specimens filled with multilayered shell–core-structured microcapsules are shown in Figure 4a,b. The parameters of the crystalline melt properties obtained are shown in Table 1, where ω is the microcapsule content within the composite, peak melting T_m_ is the temperature at the end of the melt in the heat flow change curve of the melting process, and peak crystallization T_c_ is the temperature at the moment of the maximum rate of crystallization formation in the heat flow change curve of the crystallization process. The calculation method of crystallinity is shown in Equation (4).

(4) $$X_{c}=\frac{\Delta H_{m}}{\Delta H_{100}}$$

where *X*_c_ represents the crystallinity of the composite material; Δ*H_m_* is the heat of fusion of the test sample, that is, the area surrounded by the melting peak and the baseline; Δ*H*_100_ represents the heat of fusion of an ideal material when it is 100% crystallized; and XLPE takes the value of 288 J/g.

The microcapsule system improves the crystallinity of the material. Thus, the addition of the microcapsule system can make many interface areas appear in the XLPE material. These interfacial regions in the process of recrystallization of the material play the role of heterogeneous nucleation, which improves the crystallization rate of the material and inhibits the growth rate of spherical crystals. Increasing the number of homogeneous and orderly spherical crystals within the material, so that the distribution of the crystallization size is more centralized and uniform, increases the material crystallinity. Therefore, the crystallization size distribution is more concentrated and uniform, which improves the material crystallinity. However, compared with the bilayer shell–core microcapsules, the nano-SiO_2_ on the surface of the trilayer shell–core microcapsules had a heterogeneous nucleation effect, which reduced the grain size and increased the number of grains within the material and improved the integrity of the grains. Meanwhile, the nano-SiO_2_ can make the microcapsule and the XLPE matrix have better mechanical interlocking ability, which makes the material’s internal structure tighter and inhibits the occurrence of the phenomenon of agglomeration. Therefore, the crystallinity of the XLPE specimen filled with trilayer shell–core microcapsules was slightly higher than that of the XLPE specimen filled with bilayer shell–core microcapsules.

#### 3.2.2. AC Breakdown Characteristic

**Figure 5:**
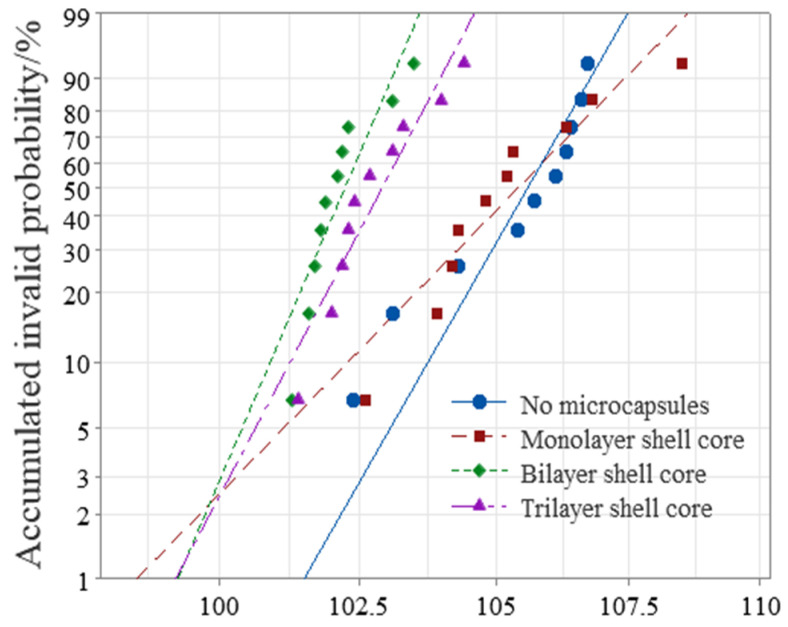
Weibull distribution of the AC breakdown field strength of the XLPE specimens filled with multilayered shell–core-structured microcapsules.

This paper used Weibull statistical distribution to process the obtained breakdown field strength data. The AC breakdown field strength test results of XLPE specimens filled with multilayered shell–core-structured microcapsules are shown in Figure 5. As it can be seen from Figure 5, the microcapsule system decreased the breakdown field strength of the XLPE materials. Furthermore, the breakdown field strength of the XLPE filled with monolayer shell–core microcapsules was close to that of the pure XLPE specimens, and that of the XLPE filled with trilayer shell–core microcapsules was slightly higher than that of the XLPE filled with bilayer shell–core microcapsules. Since the insulating strength of the microcapsules was lower than that of pure XLPE specimens, the doping of the microcapsules reduced the insulating strength of the overall XLPE specimen. Compared with the pure XLPE specimens, the incorporation of microcapsules introduced a large number of impurities that increased the carrier concentration, thereby increasing the collisional ionization under the electric field and decreasing the dielectric strength of the XLPE specimens. In addition, of the three structural microcapsules, the monolayer shell–core microcapsule had the smallest size; so, the breakdown field strength of the XLPE specimen filled with monolayer shell–core microcapsules was the highest, which was close to that of the pure XLPE specimen. Furthermore, compared with the bilayer shell–core microcapsules, the nano-SiO_2_ on the surface of the trilayer shell–core microcapsules made the grains within the XLPE material more intact, improved the crystallinity of the material, reduced the average free travel of the resistance within the material, and decreased the electron energy. It is difficult to form an electron avalanche, thereby increasing the breakdown field strength of the material. In addition, a small amount of SiO_2_ on the surface of the microcapsule can increase the conductivity of the material, improve the carrier injection barrier, introduce deep traps within the material, which have a trapping effect on the carriers, and reduce the injection of carriers. Furthermore, nano-SiO_2_ can make the microcapsules and XLPE matrix have better mechanical interlocking ability so that the internal structure of the material is more compact, weakening the electric field distortion at the location of the microcapsules and inhibiting the generation of partial discharges. Accordingly, the breakdown field strength of the XLPE specimen filled with trilayer shell–core microcapsules was slightly higher than that of the XLPE specimen filled with bilayer shell–core microcapsules.

#### 3.2.3. Dielectric Properties

**Figure 6:**
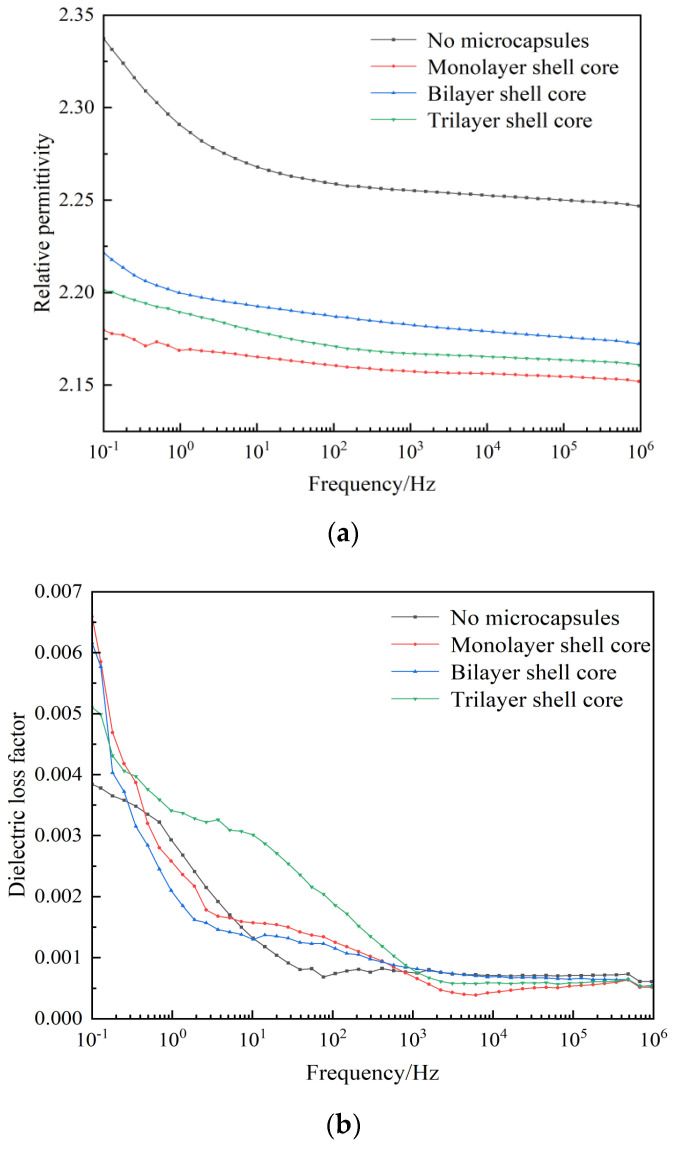
(**a**) Relative dielectric constants of the XLPE specimens filled with multilayered shell–core-structured microcapsules. (**b**) Dielectric loss factor of XLPE specimens filled with multilayered shell–core-structured microcapsules.

The relative permittivity and dielectric loss factor versus the frequency curves of XLPE specimens filled with multilayered shell–core-structured microcapsules are shown in Figure 6a,b. It can be seen from the figure that both the dielectric constant and dielectric loss factor increased with the decrease in frequency. In the low-frequency part, the electronic polarization energy within the material was synchronized with the external electric field, and the dielectric loss was dominated by the conductivity loss. However, in the high-frequency part, the dipole-steering polarization of the polar molecules within the material was difficult to synchronize with the external electric field. The relaxation polarization was hard to establish, which reduced the relaxation polarization loss of the charge traps, leading to an increase in the relative permittivity and dielectric loss factor of the material with the decrease in frequency.

In addition, the dielectric constant of the three XLPE specimens filled with multilayered shell–core-structured microcapsules was lower than that of the pure XLPE specimens, and the dielectric loss factor increased compared to that of the pure XLPE specimens. The nano-SiO_2_ on the surface of the trilayer shell–core microcapsules improved the crystallinity of the XLPE material, which resulted in a tighter structure within the material, better grains, more difficult dipole steering polarization, and reduced dielectric constant of the material. Likewise, due to the formation of interfacial domains between the nano-SiO_2_ and XLPE matrix, many deep traps were introduced inside the material, which will trap the carriers during their migration and reduce carrier mobility. The mechanical interlocking ability between the nano-SiO_2_ on the surface of the microcapsules and the XLPE matrix will make the molecular arrangement within the material more compact. The, the molecular chain flexibility is improved, the binding force between molecular chains is increased, and the electronic energy is hard to accumulate, which results in a decrease in the dielectric constant and the dielectric loss factor in the low-frequency part. In summary, the three XLPE specimens filled with multilayered shell–core-structured microcapsules showed a reduction in the relative dielectric constant and no significant change in the dielectric loss factor, which satisfied the practical application requirements of XLPE materials.

#### 3.2.4. Space Charge Characteristics

**Figure 7:**
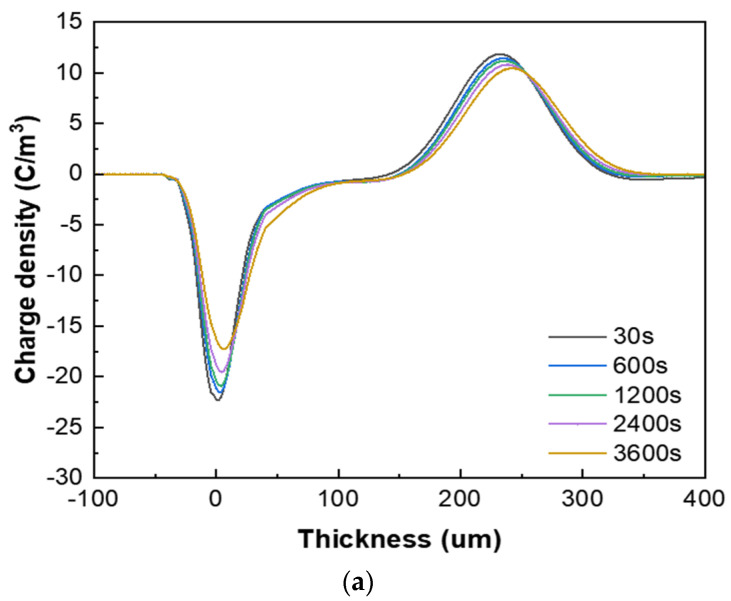

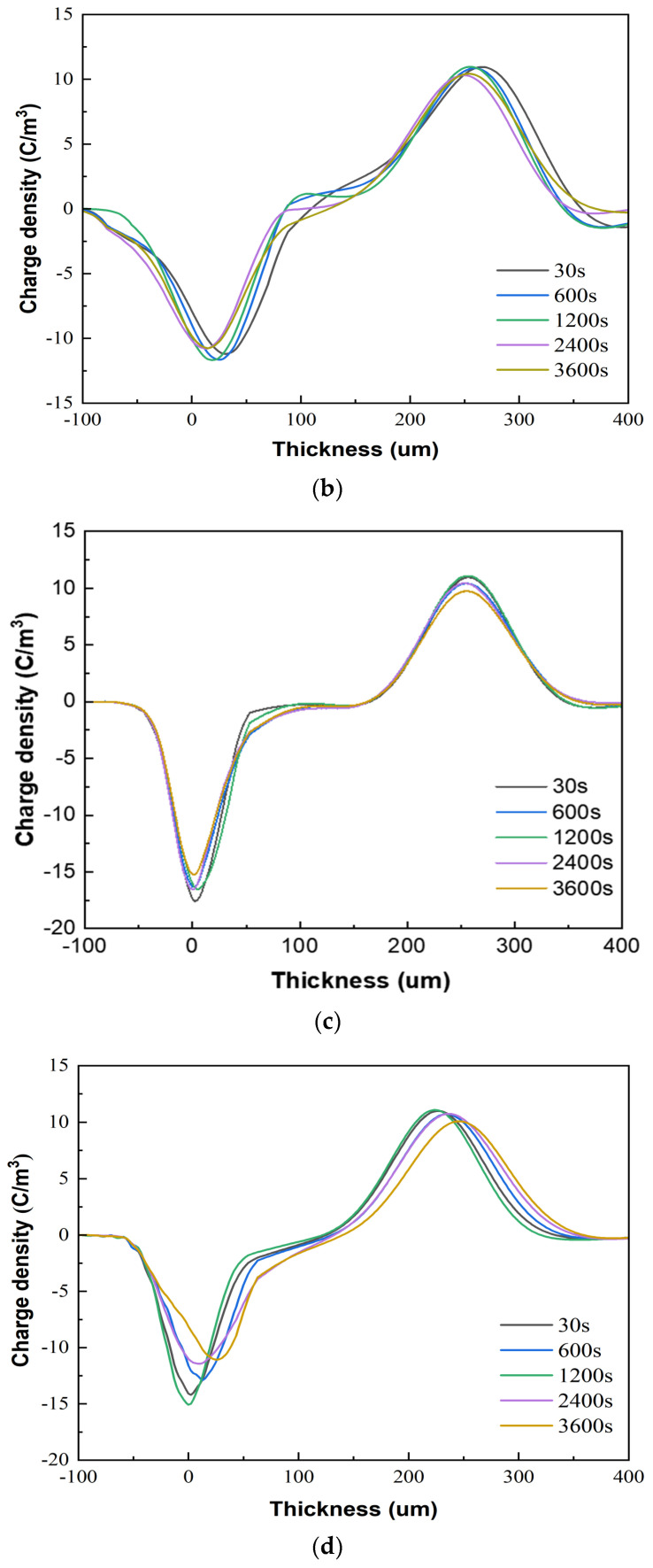
The space charge distribution map of the XLPE specimens filled with multilayered shell–core–structured microcapsules: (**a**) no microcapsules; (**b**) monolayer shell−core microcapsules; (**c**) bilayer shell−core microcapsules; (**d**) trilayer shell−core microcapsules.

Figure 7 presents the space charge test results of the XLPE specimens filled with multilayered shell–core-structured microcapsules under pressurized polarization for 3600 s at an electric field of 48 kV/mm at room temperature. For the pressurized polarization part, the acquisition times was 30 s, 600 s, 1200 s, 2400 s, and 3600 s. The space charge density of the XLPE specimens doped with microcapsules was significantly lower than that of the pure XLPE specimens. On the one hand, the XLPE specimens filled with monolayer shell–core microcapsules and bilayer shell–core microcapsules showed anisotropic charge accumulation near the cathode and homopolar charge near the anode. On the other hand, the XLPE specimen filled with trilayer shell–core microcapsules did not experience anisotropic charge accumulation near the cathode but rather a homopolar charge near the cathode and anode. There were two primary sources of space charge within the XLPE specimens. One of them was the formation of homopolar charge by the migratory carriers or incoming trapped carriers generated by the electrode injection. The other was the formation of anisotropic charge by the internal polar impurities of the material that were ionized under the action of the electric field and migrated to the vicinity of the electrode. The microcapsule system increased the number and depth of charge traps inside the XLPE material. Each microcapsule became the charge concentration area inside the material, forming a dense and uniformly distributed charge shielding layer and reducing the material’s conductivity, thus effectively shielding the charge injection phenomenon on the electrode surface and achieving the effect of inhibiting space charge injection and migration. Moreover, polar impurities were also introduced to enhance ionization and increase the internal anisotropic charge, which counteracted the space charge injected by the electrodes, thus reducing the space charge density within the material.

In addition, the nano-SiO_2_ introduced a large number of deep traps within the XLPE material, which restricted the transportation of carriers within the material and acted as a hindrance to the electrode injection charge. At the same time, the electrode injected under the action of the electric field was captured by the deep traps, forming homopolar charge accumulation near the electrode and further weakening the electric field near the electrode. Therefore, compared with the bilayer shell–core microcapsules, the nano-SiO_2_ on the surface of the trilayer shell–core microcapsules reduced the maximum space charge density of the material, and the suppression effect of space charge was more prominent.

In conclusion, the microcapsule system can improve the crystallinity of XLPE materials, inhibit carrier migration, and reduce the dielectric properties and space charge density of the materials. However, it can also produce structural defects at the interface and reduce the breakdown strength of XLPE materials. The typical properties of the XLPE specimen filled with monolayer shell–core microcapsules are close to those of the pure XLPE samples. The SiO_2_ on the surface of the trilayer shell–core microcapsules can make the microcapsules and XLPE matrix have better mechanical interlocking ability so that the internal structure of the material is more compact and the occurrence of agglomeration phenomenon is inhibited. Therefore, the typical properties of the XLPE specimen filled with trilayer shell–core microcapsules are slightly higher than those of the XLPE specimen filled with bilayer shell–core microcapsules.

### 3.3. Self-Healing Performance of the Water Tree

#### 3.3.1. Microscopic Observation of Water Tree Damage Self-Repair Ability

**Figure 8:**
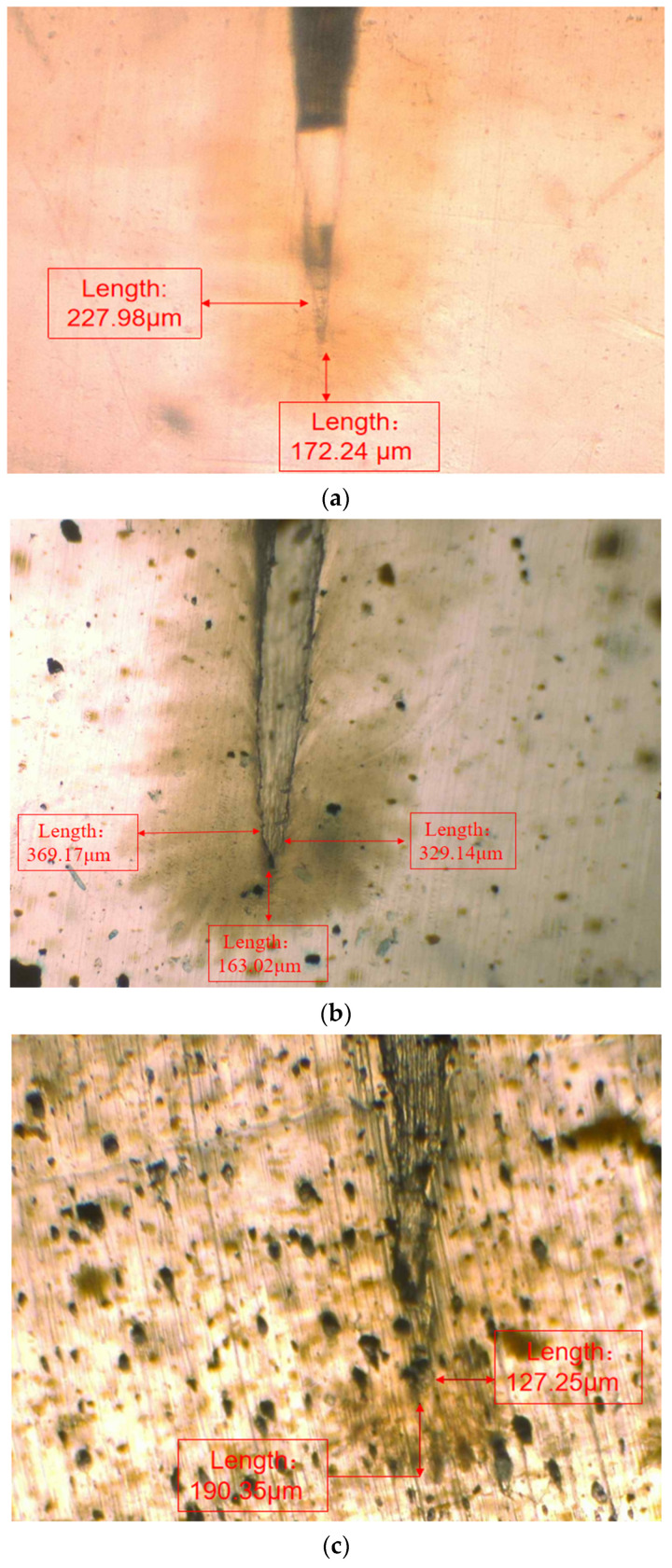

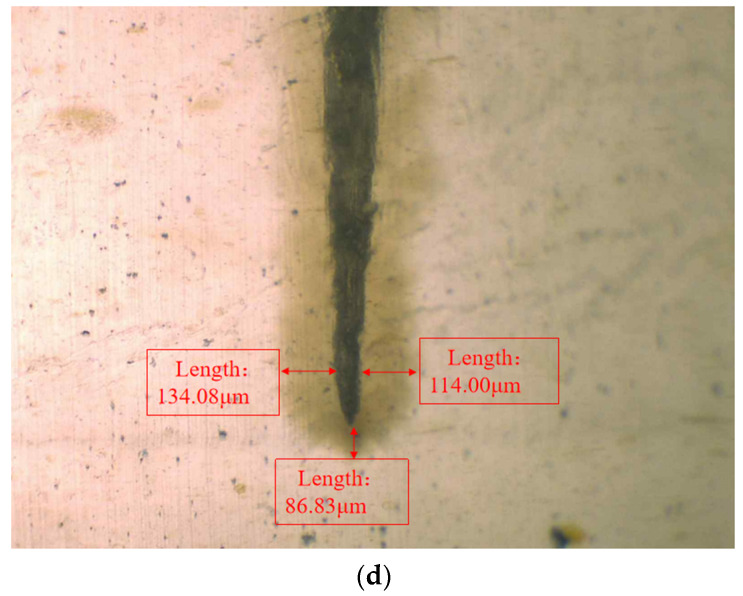
The OM microstructure of the 30-day aging water tree area of the sample: (**a**) no microcapsules; (**b**) monolayer shell–core microcapsules; (**c**) bilayer shell–core microcapsules; (**d**) trilayer shell–core microcapsules.

In order to observe the water tree growth of the XLPE specimens filled with multilayered shell–core-structured microcapsules, the samples were sliced and stained, after which the water tree morphology was observed using OM. The microscopic morphology of the water tree of XLPE specimens filled with multilayered shell–core-structured microcapsules after aging for 30 d is shown in Figure 8. It can be observed that the water tree branches tended to grow towards the presence of microcapsules. By comparing the XLPE specimen without microcapsules and those filled with monolayer shell–core, bilayer shell–core, and trilayer shell–core microcapsules, it can be seen that the water tree length of the sample doped with monolayer shell–core microcapsules was the largest, even higher than that of the pure XLPE samples. The monolayer shell–core microcapsules could not repair and fill the micropores formed in the early stage of aging due to the absence of repair material inside, which could not inhibit the growth of the water tree well. In addition, the microcapsules produced an electric field distortion inside the sample, promoting water branch development to a certain extent. Due to the presence of water tree repair material inside the bilayer shell–core and trilayer shell–core microcapsules, the microporous channels could be repaired and filled in the early stage of aging, and the growth of the water tree could be inhibited. In the meantime, when the water tree grew to the microcapsule, it broke the wall of the microcapsule and stopped growing; so, the size of the water tree was smaller than that of the pure XLPE and XLPE specimen filled with monolayer shell–core microcapsules. As it can be seen from the OM plots of the water tree of the XLPE specimens filled with bilayer shell–core and trilayer shell–core microcapsules, the length of the water tree of the specimens doped with trilayer shell–core microcapsules decreased further. The nano-SiO_2_ on the surface of the trilayer shell–core microcapsules made the microcapsules have a better mechanical interlocking ability with the XLPE matrix. It made the internal structure of the material more compact, weakened the electric field distortion at the location of the microcapsules, and prevented the occurrence of agglomeration. Therefore, it had a better effect on inhibiting water tree growth.

**Figure 9:**
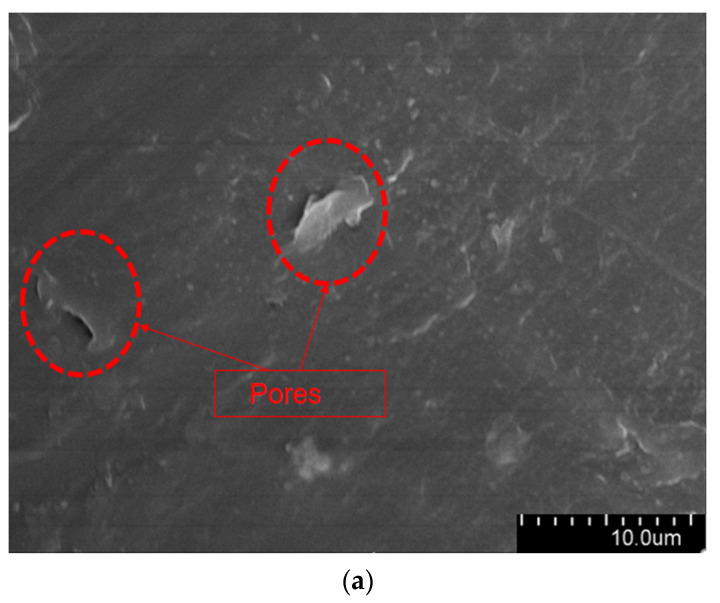

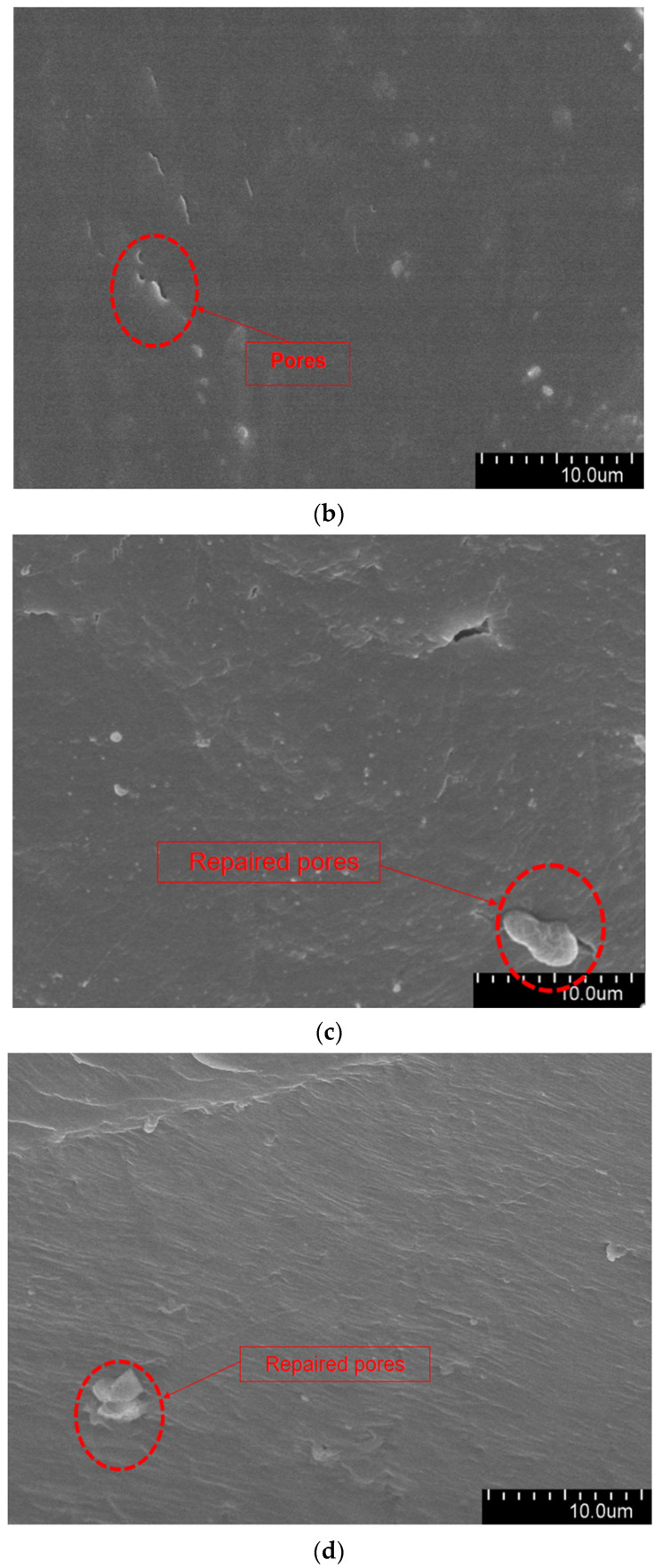
The SEM observation results of the 30-day aging water tree area of the sample: (**a**) no microcapsules; (**b**) monolayer shell–core microcapsules; (**c**) bilayer shell–core microcapsules; (**d**) trilayer shell–core microcapsules.

The aged XLPE specimens filled with microcapsules were quenched at the tip of the needle and subjected to SEM observation. Figure 9 shows the micromorphology of the water tree region. As it can be seen from the Figure 9, the XLPE specimens subjected to water tree aging produced a series of holes, and these microscopic holes are also typical of water tree generation. Furthermore, compared with the pure XLPE specimens and XLPE specimen filled with monolayer shell–core microcapsules, some of the holes in the XLPE specimens containing bilayer shell–core or trilayer shell–core microcapsules were filled. It was proved that, after water tree aging, the local high electric field broke the bilayer shell–core or trilayer shell–core microcapsules. At the same time, the repair material reacted with water to generate silicone resin to fill the holes, thus achieving the self-repairing ability of the material.

**Figure 10:**
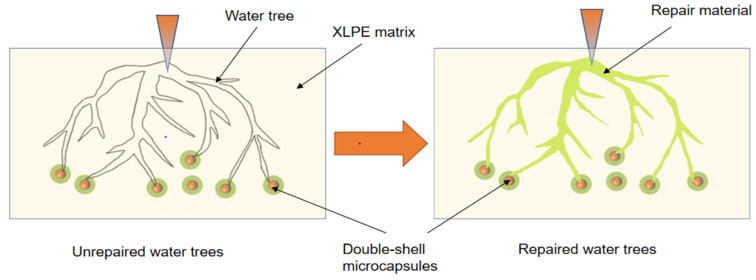
Schematic diagram of the water tree aging self-healing principle.

**Figure 11:**
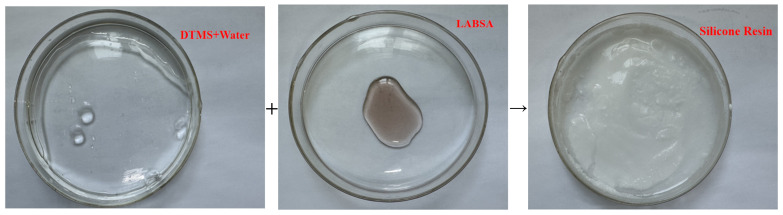
Reaction of the repair solution with water at room temperature.

The water tree aging self-healing mechanism of the XLPE specimens filled with microcapsules is shown in Figure 10. Based on the images, it can be observed that water tree aging is more likely to occur in the region where microcapsules exist due to impurities and structural defects. Meanwhile, because of the difference in dielectric constant between the microcapsules and XLPE specimens, the interface between the two is prone to showing the presence of polarized charge accumulation, which improves the local electric field between the water needles and the microcapsules, controlling the way the water tree grows. Therefore, when the water tree develops to the microcapsule, it consumes a lot of energy by breaking the outer and inner capsule walls of the microcapsules sequentially, which hinders the further growth of the water tree. Simultaneously, the bilayer shell–core or trilayer shell–core microcapsules rupture under the electric field, and the repair liquid and catalyst are mixed by capillary action into the water tree damage area. Under the action of the catalyst, the siloxane repair solution in the microcapsules undergoes hydrolysis and condensation reaction with water, generating a new silicone oxygen group organic polymer silicone resin and methanol. The primary chemical equations for the reaction are shown in Equations (5) and (6). The reaction of the siloxane repair solution DTMS with water under the action of the catalyst at room temperature is shown in Figure 11.

(5) $${\mathrm{PhMeSi}(\mathrm{OMe})}_{2}{+H}_{2}O\to \mathrm{PhMeSi}(\mathrm{OH})(\mathrm{OMe})+\mathrm{MeOH}$$

(6) $${\mathrm{PhMeSi}(\mathrm{OH})(\mathrm{OMe})+H}_{2}O\to {\mathrm{PhMeSi}(\mathrm{OH})}_{2}+\mathrm{MeOH}$$

where Me and Ph are methyl and phenyl, respectively; and X is the number of moles. The reaction consumes the water in the composite material, and the organic polymer generated after the reaction fills the tiny pores, thereby eliminating the water tree and restoring the insulating properties of the water tree-aged specimen. The organic silicone resin is not easy to decompose at high temperatures. It not only has the characteristics of high-pressure resistance and better insulation performance but also its performance is similar to that of XLPE. Its dielectric constant is 2.6~2.8, close to that of the XLPE material (ε_r_ ≈ 2.26) and far lower than that of the water tree area before repair. In addition, the conductivity is below 10^−9^ S/m, which is lower than that of the water tree area before repair (γ ≈ 5 S/m), and effectively fills the hollow of the water tree.

#### 3.3.2. PDC Analysis of the Water Tree Aging Sample

To further verify the self-healing effect of the material, the unaged XLPE specimen not filled with microcapsules, the unaged XLPE specimen filled with bilayer shell–core microcapsules, the unaged XLPE specimen filled with trilayer shell–core microcapsules, the aged XLPE specimen not filled with microcapsules, the aged XLPE specimen filled with bilayer shell–core microcapsules, and the aged XLPE filled with trilayer shell–core microcapsules were tested using PDC, so as to obtain six groups of different experimental data. The obtained depolarization current and the corresponding time relationship were recorded and plotted as images.

**Figure 12:**
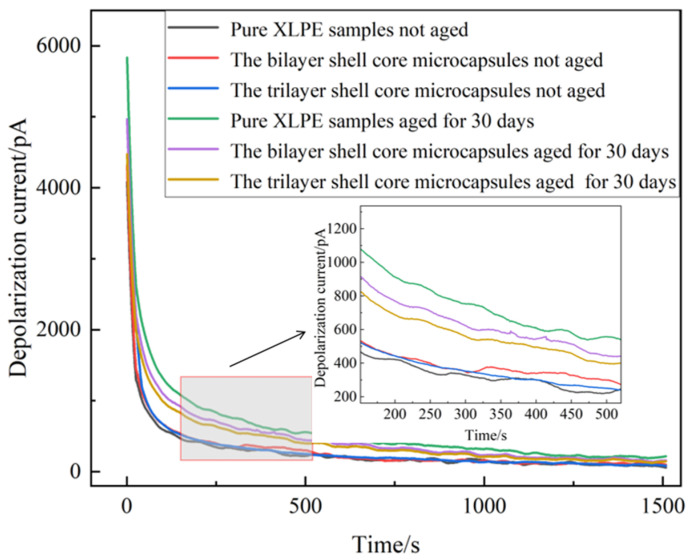
Depolarization current of XLPE specimens filled with microcapsules.

The depolarization current images obtained from the tests are shown in Figure 12. It can be seen that the onset and stable values of the depolarization current of the pure XLPE specimen and the XLPE specimens filled with microcapsules increased significantly after the water tree aging test. This indicates that the XLPE samples underwent water tree aging with increased internal traps and enhanced polarization characteristics. The water tree aging defect accelerated the degree of insulation degradation of the material, leading to a decrease in the insulation strength of the XLPE specimens. Comparing the unaged pure XLPE specimens with the unaged XLPE specimens filled with microcapsules, there was a slight increase in the depolarization current of the material after doping with the microencapsulated system. Thus shows that the microcapsule system aggravated the interfacial polarization process of the material. However, when the microcapsule doping content was 1.0 wt%, the change in depolarization current was small and had little effect on the initial properties of the specimens.

The XLPE specimens filled with bilayer shell–core microcapsules and trilayer shell–core microcapsules were compared. Both before and after aging, the depolarization current test results of the XLPE specimen filled with trilayer shell–core microcapsules were better than those of the XLPE specimen filled with bilayer shell–core microcapsules, which is in line with the results of the experimental tests in the above sections. This shows that the nano-SiO_2_ on the surface of the trilayer shell–core microcapsules can effectively improve the various properties of the composites. However, compared to the pure XLPE specimen, the XLPE specimen doped with microcapsules had lower onset and stable values of depolarization current after water tree aging. This shows that the microcapsule system can repair the water tree damage area within the material, fill the tiny holes formed after aging, and reduce the degradation degree of the material. At the same time, the generated organic polymer has similar electrical properties to the matrix, which can reduce the internal polarization current and restore the insulating properties of the material. Since the microcapsules cannot completely repair the tiny defects formed by aging, their properties cannot be restored to the initial state of the material, but they still show a good self-healing ability.

#### 3.3.3. Simulation Analysis of Electric Field Distribution

**Figure 13:**
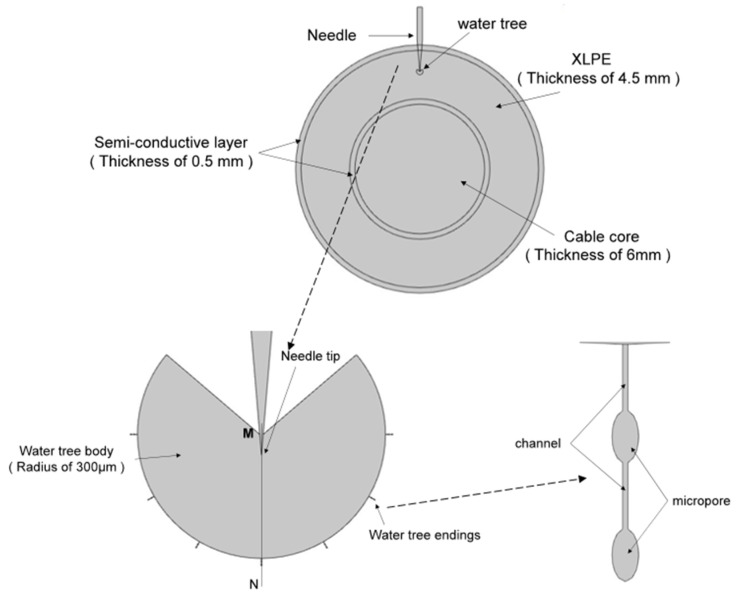
The simulation model of the water tree area.

To analyze the electric field distribution of the samples before and after water tree self-healing, the water tree aging model of the XLPE cable was established using multi-physical field finite element simulation software. The simulation model is shown in Figure 13. In addition, Table 2 shows the parameters of the materials used in the simulation model. The water tree part consisted of a 260° fan-shaped main body and seven tips characterized by “strings of pearls”. The channels at the end of the water tree were 5 μm long and 0.2 μm wide, and the micropores had a long axis of 4 μm and a short axis of 2 μm.

**Figure 14:**
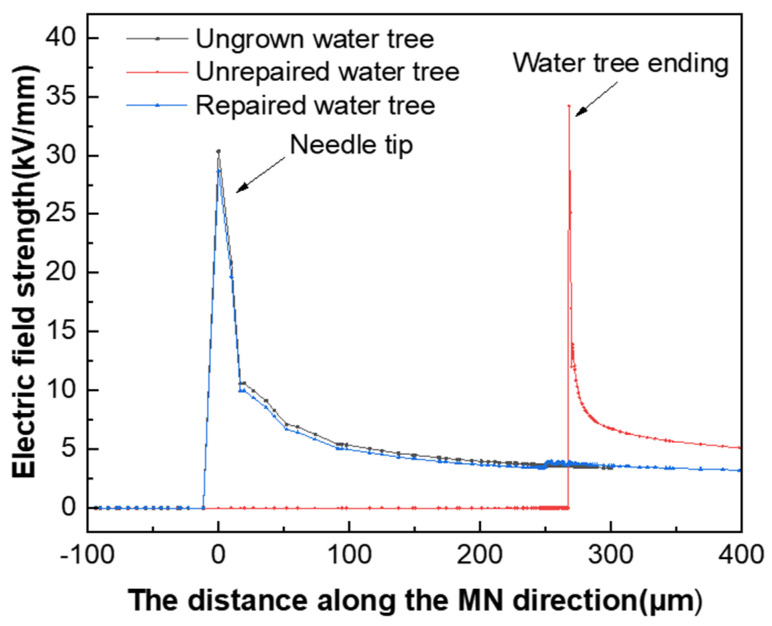
Electric field distribution of the water tree area before and after repair.

To investigate the effect of the presence of the water tree on the electric field distribution in the tip region, the electric field distribution in the tip area was simulated and calculated when the water tree was repaired and when the water tree was not repaired. Figure 14 presents the simulation results of the electric field distribution along the direction of the needle tip and the end of the water tree. From Figure 14, it can be seen that the maximum electric field strength of the material was 34 kV/mm when the water tree was present within the material, which was located at the end region of the water tree. When the maximum electric field strength of the material was reduced to 28 kV/mm after the water tree repair, the location changed from the end region of the water tree to the tip of the water needle. Since the electrical properties of the silicone resin generated after water tree repair were close to those of the XLPE substrate, it effectively filled the XLPE matrix. In addition, it weakened the electric field in the water tree area, which made the electric field distribution of the material match that of the material when the water tree did not grow.

## 4. Conclusions

In this paper, the influence of the structure of microcapsules on the properties of XLPE composites was investigated. Three kinds of microcapsule samples with multilayered shell–core structures were innovatively designed. By preparing XLPE specimens filled with multilayered shell–core-structured microcapsules, the influence of microcapsule system on the typical properties of XLPE materials was analyzed. At the same time, the self-healing ability of the material was verified. The main conclusions are as follows:

(1) The microcapsules improved the crystallinity of the material through heterogeneous nucleation with the XLPE matrix and improved the electrical properties of the material by reducing characteristics such as carrier migration and material polarization.
(2) Microcapsules can control the growth pattern of water trees. Furthermore, the rupture of the microcapsules depletes the energy of the water tree and inhibits the developmental stage of the water tree.
(3) The SiO_2_ on the surface of the trilayer shell–core microcapsules can make the microcapsules and XLPE matrix have a better mechanical interlocking ability, which makes the internal structure of the material more compact and inhibits the occurrence of agglomeration phenomenon. Therefore, the typical properties of the XLPE specimen filled with trilayer shell–core microcapsules were slightly higher than those of the XLPE specimen filled with bilayer shell–core microcapsules.
(4) When water tree aging occurred in the XLPE specimens filled with bilayer shell–core microcapsules and trilayer shell–core microcapsules, the outer and inner capsule walls of the microcapsules ruptured, and the repair material reached the water tree aging area. It reacted with water to consume it inside the XLPE material and fill the micropores in the water tree aging area. The generated organic matter repairs the water tree aging area of the material restored the insulating properties of the material and improved the negative impact of insulation aging on the material. This has a good research value for practical applications.

## Figures and Tables

**Table 1:** Thermal parameters of the melting–crystallization characteristics and the calculated crystallinities.

Microcapsule Structure/%	Peak Melting *T*_m_/°C	Crystallization Peak *T*_c_/°C	Crystallinity *X*_c_/%
No microcapsules	107.6	94.6	33.73
Monolayer shell–core microcapsules	109.1	95.6	38.92
Bilayer shell–core microcapsules	107.1	94.3	36.43
Trilayer shell–core microcapsules	109.5	94.2	37.31

**Table 2:** Parameters of the simulation model.

Component	*ε*_r_	*γ*/(S/m)
XLPE	2.3	1 × 10^−17^
Cable core	1 × 10^6^	5.98 × 10^7^
Semi-conductive layer	100	1 × 10^−3^
Unrepaired water tree area	16	1 × 10^−7^
Repaired water tree area	2.65	1 × 10^−11^

## Data Availability

Data are contained within the article.
